# Treatment of infected thoracic aortic aneurysm with combined abscess debridement and stent-graft wrapping using pedicled latissimus dorsi muscle flaps after thoracic endovascular aortic repair

**DOI:** 10.1186/s13019-023-02155-y

**Published:** 2023-02-02

**Authors:** Hirokazu Matsushima, Tohru Ishimine, Naoki Taniguchi, Toshiho Tengan

**Affiliations:** Department of Cardiovascular Surgery, Okinawa Chubu Hospital, 281, Miyazato, Uruma-Shi, Okinawa 904-2293 Japan

**Keywords:** Infected thoracic aortic aneurysm, Thoracic endovascular repair, Latissimus dorsi muscle flaps, Stent-graft wrapping

## Abstract

**Background:**

Open thoracic surgery (with infected lesion removal, prosthetic graft replacement, and pedicled tissue flap) has remained the main treatment for infected thoracic aortic aneurysms to date. Recent reports have highlighted good prognostic outcomes with thoracic endovascular aortic repair. However, thoracic endovascular aortic repair for infected thoracic aortic aneurysms is associated with an exacerbation of infection due to residual infected tissues. We discuss the control of refractory infections following endovascular treatment of infected thoracic aortic aneurysms.

**Case presentation:**

An 81-year-old man, with a history of insulin-dependent diabetes mellitus and pancreaticoduodenectomy, presented to our emergency department with a fever. Blood tests revealed a markedly elevated leukocyte count, and contrast-enhanced computed tomography suggested a descending thoracic aortic pseudoaneurysm. We diagnosed the patient with an infected descending thoracic aortic aneurysm, and performed urgent thoracic endovascular aortic repair; he was started on an intravenous antibiotic treatment. Postoperatively, blood tests revealed a decreased leukocyte count and the patient remained afebrile. However, computed tomography revealed temporal enlargement of the abscess cavity; therefore, an abscess debridement and stent graft wrapping with pedicled latissimus dorsi muscle flaps were performed, which successfully controlled the infection. Six weeks after abscess debridement, the patient was switched to an oral antibiotic therapy. There was no evidence of recurrence of infection 8 months after the surgery.

**Conclusions:**

A combined abscess debridement and pedicled tissue flap approach is useful for patients with poor surgical tolerance in whom infection control is difficult after thoracic endovascular aortic repair for infected thoracic aortic aneurysms. Pedicled latissimus dorsi muscle flaps are useful when using the omentum for pedicled tissue flap is difficult.

## Background

Patients with infected thoracic aortic aneurysms (ITAAs) have a poor prognosis and are at a high risk of rupture. Additionally, the incidence of sepsis-led multiorgan failure and the mortality rate in this population are high. Although open thoracic surgery (with infected lesion removal, prosthetic graft replacement, and pedicled tissue flap) has remained the main treatment for ITAAs until now, recent reports have highlighted good prognostic outcomes with thoracic endovascular aortic repair (TEVAR) [1]. However, TEVAR for ITAAs is associated with an exacerbation of infection due to residual infected tissues. Herein, we have reported a case wherein infection control was difficult to achieve after TEVAR for a ruptured ITAA; the patient was successfully managed using abscess debridement and stent graft wrapping with pedicled latissimus dorsi muscle (LDM) flaps instead.

## Case presentation

**Fig. 1:**
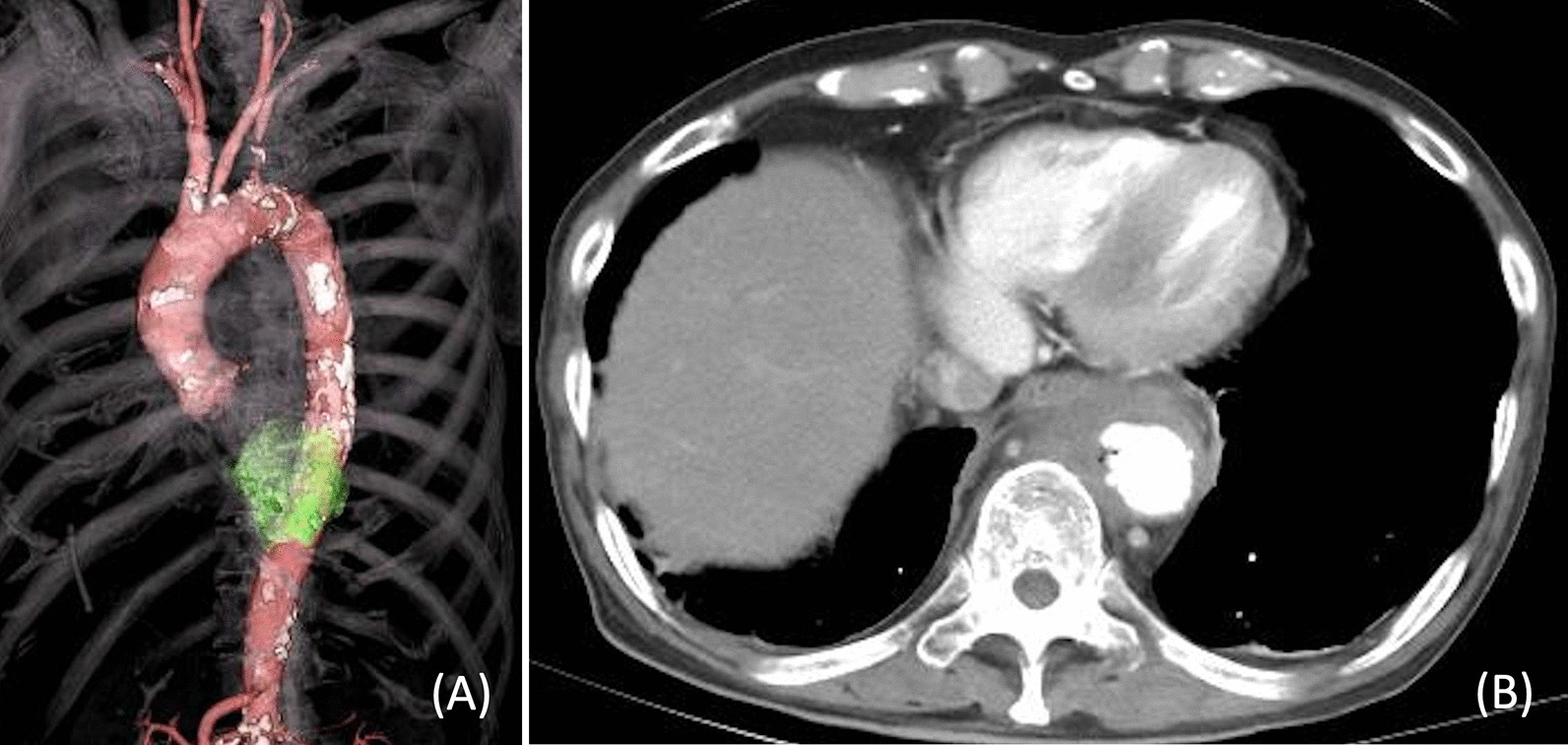
Computed tomography (CT) before the first thoracic endovascular aortic repair (TEVAR) reveals a ruptured pseudoaneurysm in the descending thoracic aorta (at the level of Th10) surrounded by fluid accumulation (**A**: 3D CT; **B**: contrast-enhanced CT)

An 81-year-old man, with a 3-day history of loss of appetite and abdominal pain, presented to the emergency department of our hospital with fever and tachypnoea. His hemodynamic status was stable; however, chills, shivering, and mild tenderness in the upper abdomen were observed at presentation. The patient had a history of hypertension, hyperlipidaemia, insulin usage for type 2 diabetes mellitus, percutaneous coronary intervention for unstable angina pectoris, and postoperative lobectomy for right lung cancer. The patient had also undergone pancreaticoduodenectomy for a duodenal papillary carcinoma and had experienced recurrent cholangitis since then. Blood tests revealed a white blood cell count and C-reactive protein level of 12,100/µL and 10.87 mg/dL, respectively. While chest radiography revealed no abnormalities, contrast-enhanced computed tomography (CT) revealed a pseudoaneurysm in the descending thoracic aorta (at the level of Th10) surrounded by fluid accumulation (Fig. 1A, B). Based on the fever, inflammatory response, and the aneurysm shape, we diagnosed the patient with ITAA.

**Fig. 2:**
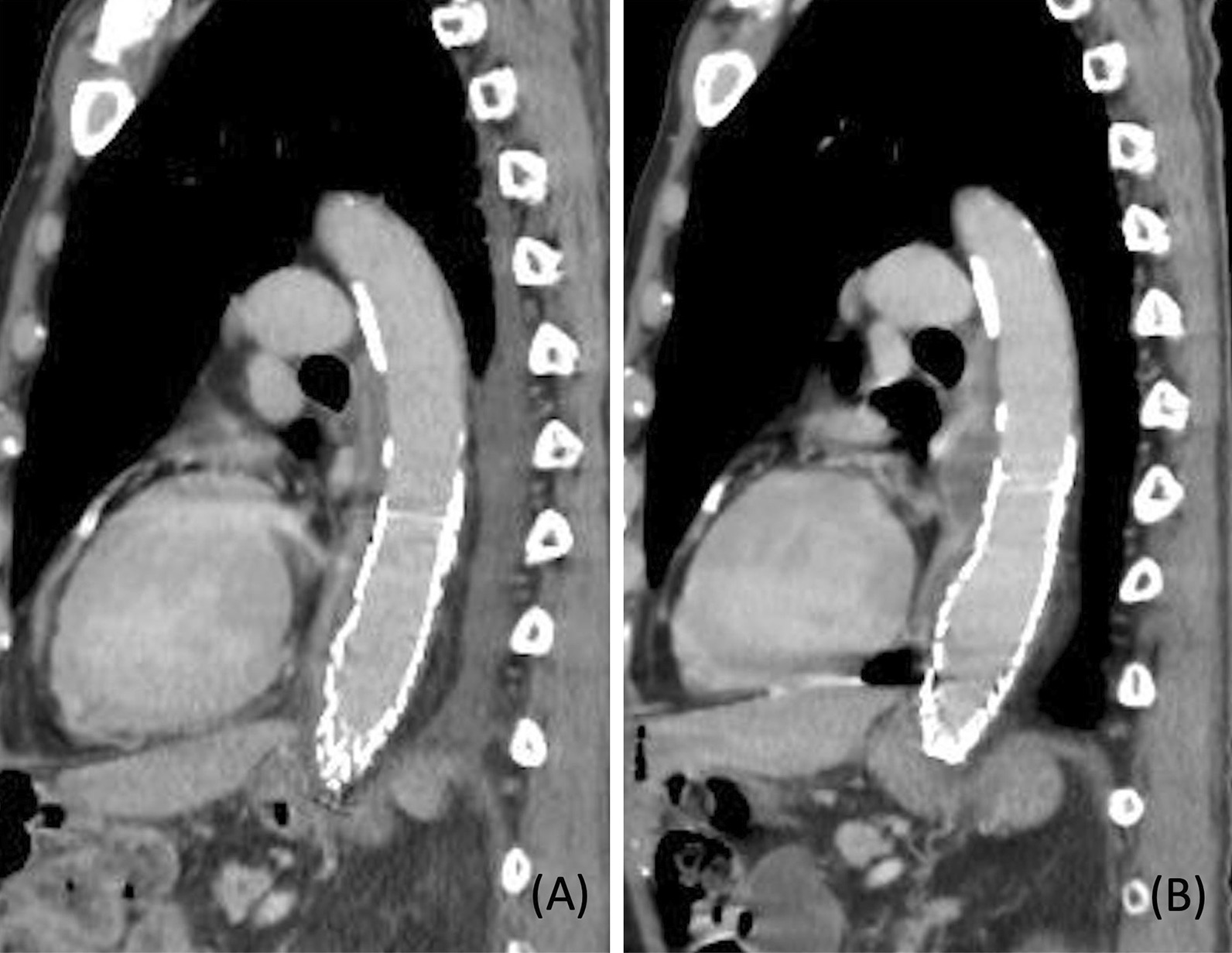
CT performed 17 days after TEVAR revealed that the abscess cavity had extended beyond the proximal edge from the implanted stent graft, i.e., in the area uncovered by the graft (**A**: three days after the initial TEVAR; **B**: 17 days after the initial TEVAR)

**Fig. 3:**
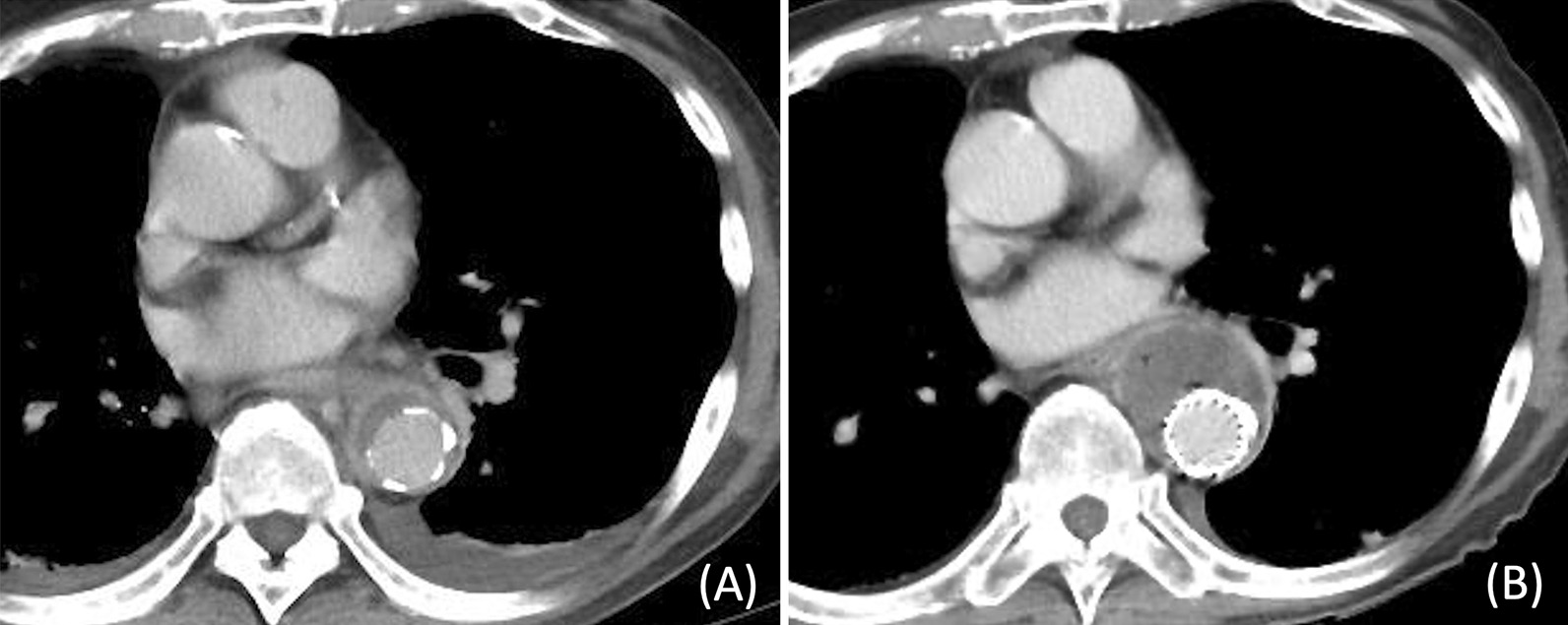
Despite antibiotic treatment after TEVAR, contrast-enhanced CT suggests abscess expansion over time (**A**: three days after the initial TEVAR; **B**: 29 days after the initial TEVAR)

**Fig. 4:**
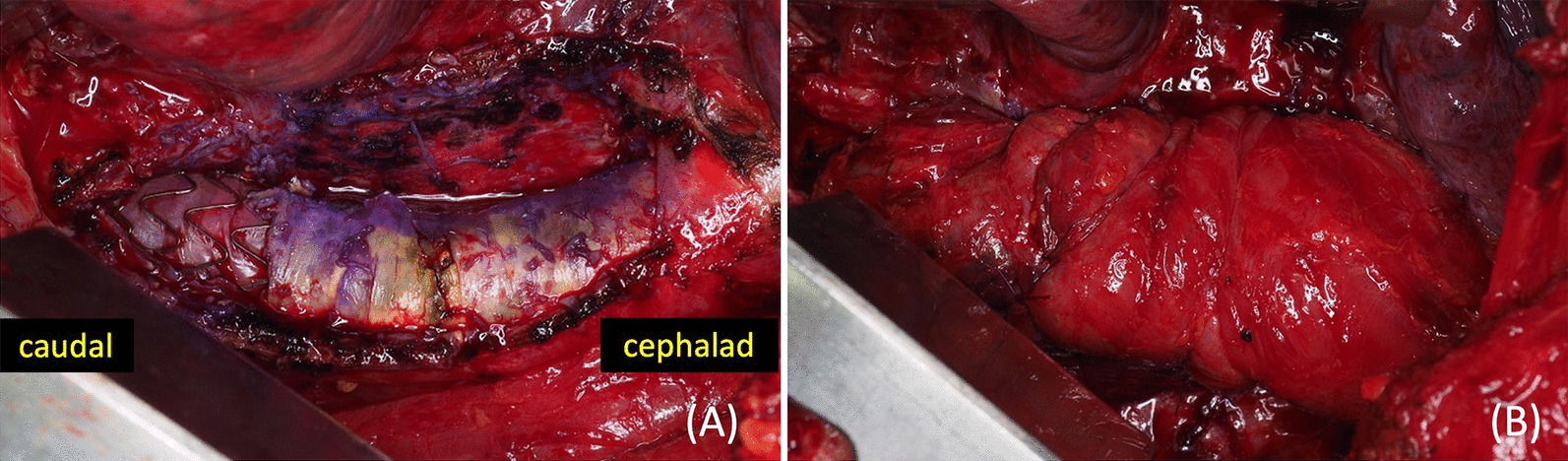
Operative image of stent graft wrapping using latissimus dorsi muscle (LDM) Flaps. The stent graft is partially exposed when the abscess cavity is released (**A**). The LDM flaps are guided into the thoracic cavity from the second intercostal space and wrapped around the stent graft (**B**)

The patient presented with a pseudoaneurysm and a localised rupture. It was feared that this would progress to a free rupture during the period of intravenous antibiotics and we decided that urgent surgical treatment was necessary. Due to his advanced age and medical history (especially insulin usage for type 2 diabetes mellitus), we determined that he would have a low tolerance for prosthetic graft replacement and performed TEVAR as an emergency procedure. Meropenem and vancomycin were administered intravenously before the TEVAR procedure. If a stent graft was placed extensively on the proximal side, there would be an increased risk of paraplegia. Therefore, a 28 × 100 mm GORE TAG device (W. L. Gore and Associates, Flagstaff, Ariz) was implanted during the procedure to cover the aortic aneurysm protrusion (6 cm proximally from the protrusion and 4 cm distally from the protrusion). One week after TEVAR, a blood culture taken before TEVAR yielded *Escherichia coli,* so the antibiotic therapy was switched to cefotaxime. The inflammatory response decreased, and the patient remained afebrile; however, CT performed 17 days after TEVAR revealed that the abscess cavity had extended beyond the proximal edge (3.5 cm from the implanted stent graft), i.e., in the area uncovered by the graft (Fig. 2A, B). The aortic wall had become fragile due to infection in this uncovered area and was at risk of rupture. Considering the need for a sufficient landing zone, we implanted a 31 × 150 mm GORE TAG device (W. L. Gore and Associates, Flagstaff, Ariz) on the proximal side with a 3 cm overlap to the previous stent graft. Furthermore, considering the possibility that the patient was refractory to cefotaxime, the antibiotic therapy was switched to meropenem. However, the abscess cavity continued to enlarge (Fig. 3A, B), and we decided to perform a surgical intervention. The patient was re-evaluated for his eligibility for a prosthetic graft replacement; however, the procedure was deemed too invasive due to the initial preoperative risk as well as the patient's worsening frailty. Instead, abscess debridement and pedicled tissue flap were performed for further infection control. The use of the omentum for the pedicled tissue flap was deemed difficult owing to the patient’s history of pancreatoduodenectomy; thus, LDM flaps were selected instead. Preoperative CT showed that on the proximal side, the upper edge of the stent graft was 6.5 cm from the upper edge of the abscess (17.5 cm from the aortic aneurysm protrusion), and on the distal side, the lower edge of the stent graft was 3.5 cm from the lower edge of the abscess (4.5 cm from the aortic aneurysm protrusion). We also confirmed from the preoperative CT that there was no endoleak in the aortic aneurysm and no blood flow from the branch arteries into the aortic aneurysm. Based on the above, because　there was sufficient landing zone and no blood flow into the aortic aneurysm, we judged that the risk of bleeding was low. Eighteen days after the second TEVAR, a thoracoscopic abscess debridement was performed to prevent damage to the LDMs intended for use in the pedicled tissue flap. This operation was performed through a small incision made 10 cm away from the left fifth intercostal space. The abscess was located in a sub-adventitial space using ultrasound, and a 5 cm long-axis incision was made through the aortic adventitia. The abscess was debrided and its cavity was cleaned; following this, drains were placed. Similar to the blood culture, a pus culture also yielded *Escherichia coli*. Seven days after abscess debridement, stent graft wrapping was performed using LDM flaps constructed with the thoracodorsal artery as the feeding vessel. The surgery was performed through thoracotomy at the left fifth intercostal space. We widened the previously debrided aortic exenteration site in a cephalocaudal direction and completely unroofed the abscess cavity. The stent graft was partially exposed (Fig. 4A), and the abscess cavity was thoroughly flushed. The LDM flaps were guided into the thoracic cavity from the second intercostal space and wrapped around the stent graft (Fig. 4B). One week after the stent graft wrapping procedure, debridement of necrosis skin and wound closure were performed using a rectus abdominis musculocutaneous flap due to skin necrosis in the area where the LDM was harvested. Two weeks after the stent graft wrapping procedure, the antibiotic therapy was switched from meropenem to cefotiam based on the pus culture results; it was administered intravenously for 6 weeks after abscess debridement. The patient was then switched to oral levofloxacin, and a lifelong oral antibiotics policy was adopted.

**Fig. 5:**
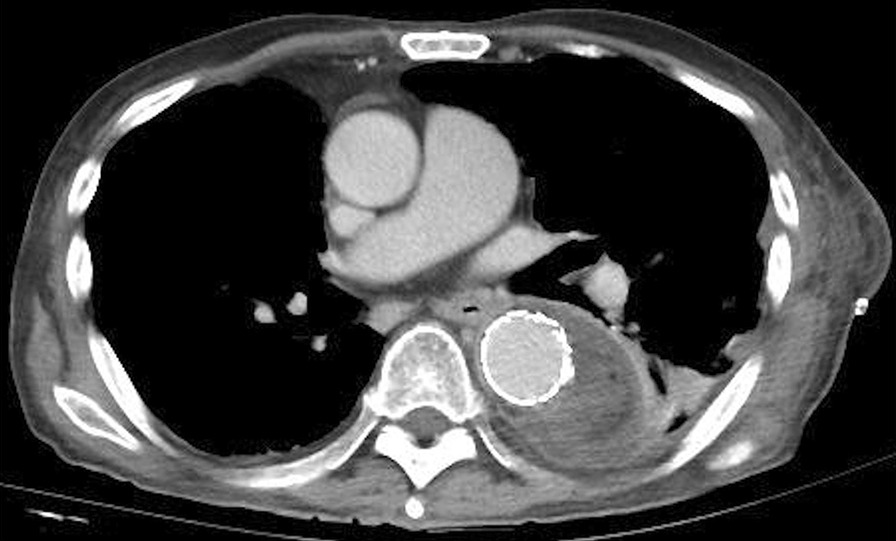
CT performed 7 days after stent graft wrapping confirmed that the LDM flaps were wrapped around the stent graft and that there was no bleeding or other complications

Postoperative CT confirmed that the LDM flaps were wrapped around the stent graft and that there was no bleeding or other complications (Fig. 5). The patient made an excellent recovery and was discharged home on day 77. No recurrent infection was observed at the 8-month outpatient follow-up.

## Discussion and conclusions

If the aortic aneurysm is stable, intravenous antibiotic treatment before surgical treatment is ideal, and surgical intervention should be performed after the infection has improved. However, the patient presented with a pseudoaneurysm and localised rupture, leading to fears of rupture during the period of intravenous antibiotics. We, therefore, performed TEVAR as an emergency procedure. During TEVAR for ITAAs, the infected tissue is not resected, and postoperative complications (including sepsis, graft infection, recurrent ITAAs, and aorto-oesophageal fistulas) have been reported in 17.3% of the cases (fatal infection complication rate: 11.5%) [1]. Nakajima et al. reported a good prognosis with abscess debridement after TEVAR for an ITAA [2]; however, one of their five patients required prosthetic graft replacement because the infection was not controlled with abscess debridement after TEVAR [2]. Therefore, we decided to perform a pedicled tissue flap in addition to abscess debridement for infection control. In our case, the patient underwent abscess debridement 18 days after the second TEVAR in the hopes that intravenous antibiotic treatment would improve the infection. In retrospect, it may have been better to debride the abscess immediately after the second TEVAR because we thought that the infection was not under control at this time.

Usually, the omentum is used for the filling procedure; however, this was difficult in our case, because the patient had a history of pancreaticoduodenectomy. LDM flaps reportedly afford a good prognosis in cases of prosthetic graft replacement for ITAAs and stent graft infection after TEVAR [3, 4]. Therefore, we performed a procedure in which the stent graft was wrapped with LDM flaps, and this is the first report of this procedure. The LDM has a fairly large area and can be filled to a level just above the diaphragm, with a margin of safety. The patient could perform daily activities without any difficulty.

When infection is difficult to control with antibiotic treatment after TEVAR for ITAAs, this strategy (abscess debridement and pedicled tissue flap) or prosthetic graft replacement are possible treatment options. Prosthetic graft replacement has the advantage of removing the stent graft and the infection adequately. However, there are risks associated with the use of cardiopulmonary bypass and heparinisation, and bleeding from the aortic anastomotic site, which can lead to postoperative complications in high-risk patients. On the other hand, our strategy requires a thoracotomy procedure but does not require cardiopulmonary bypass, heparinisation, or aortic anastomosis, and is, therefore, considered less invasive than prosthetic graft replacement. However, there is a risk of intraoperative bleeding from the aorta and stent graft sealing or from branch arteries. If bleeding occurred intraoperatively from the sealing, banding of the proximal and distal landing aorta would be performed, while if bleeding occurred from branch arteries, arterial ligation or clipping would be performed. Additionally, if the bleeding was still difficult to stop, we were prepared to perform prosthetic graft replacement. Since the stent graft is left in its place, the possibility of recurrent infection is a concern. In addition, there are risks of migration and endoleaks. Therefore, our strategy in this case requires long-term follow-up. Although it was not done in this case, we believe that banding of the proximal and distal landing aorta could prevent migration and endoleaks.

In conclusion, a combined abscess debridement and pedicled tissue flap approach is useful for patients with poor surgical tolerance in whom infection control is difficult after TEVAR for ITAAs. LDM flaps are useful when the omentum is difficult to use for pedicled tissue flap.

## Data Availability

Data sharing is not applicable to this article as no datasets were generated or analysed during the current study.

## Abbreviations

ITAAs: Infected thoracic aortic aneurysms
TEVAR: Thoracic endovascular aortic repair
LDM: Latissimus dorsi muscle
CT: Computed tomography
